# Muscle Cross-Sectional Area Segmentation in Transverse Ultrasound Images Using Vision Transformers

**DOI:** 10.3390/diagnostics13020217

**Published:** 2023-01-06

**Authors:** Sofoklis Katakis, Nikolaos Barotsis, Alexandros Kakotaritis, Panagiotis Tsiganos, George Economou, Elias Panagiotopoulos, George Panayiotakis

**Affiliations:** 1 Electronics Laboratory, Department of Physics, University of Patras, 26504 Patras, Greece; 2 Department of Medical Physics, School of Medicine, University of Patras, 26504 Patras, Greece; 3 Clinical Radiology Laboratory, School of Medicine, University of Patras, 26504 Patras, Greece; 4 Orthopaedic and Rehabilitation Department, Patras University Hospital, 26504 Patras, Greece

**Keywords:** deep learning, vision transformers, cross-sectional area, ultrasound, textural analysis

## Abstract

Automatically measuring a muscle’s cross-sectional area is an important application in clinical practice that has been studied extensively in recent years for its ability to assess muscle architecture. Additionally, an adequately segmented cross-sectional area can be used to estimate the echogenicity of the muscle, another valuable parameter correlated with muscle quality. This study assesses state-of-the-art convolutional neural networks and vision transformers for automating this task in a new, large, and diverse database. This database consists of 2005 transverse ultrasound images from four informative muscles for neuromuscular disorders, recorded from 210 subjects of different ages, pathological conditions, and sexes. Regarding the reported results, all of the evaluated deep learning models have achieved near-to-human-level performance. In particular, the manual vs. the automatic measurements of the cross-sectional area exhibit an average discrepancy of less than 38.15 mm^2^, a significant result demonstrating the feasibility of automating this task. Moreover, the difference in muscle echogenicity estimated from these two readings is only 0.88, another indicator of the proposed method’s success. Furthermore, Bland–Altman analysis of the measurements exhibits no systematic errors since most differences fall between the 95% limits of agreements and the two readings have a 0.97 Pearson’s correlation coefficient (*p* < 0.001, validation set) with ICC (2, 1) surpassing 0.97, showing the reliability of this approach. Finally, as a supplementary analysis, the texture of the muscle’s visible cross-sectional area was examined using deep learning to investigate whether a classification between healthy subjects and patients with pathological conditions solely from the muscle texture is possible. Our preliminary results indicate that such a task is feasible, but further and more extensive studies are required for more conclusive results.

## 1. Introduction

Musculoskeletal ultrasound (MSK-US) imaging is a valuable diagnostic tool for the examination of the musculoskeletal system since it enables real-time and non-invasive investigation of muscles in both normal and pathological conditions. An important application in clinical practice is the measurement of the visible cross-sectional area (CSA) since it quantifies the muscle’s size and echogenicity. These two basic architectural parameters are closely correlated with the maximal force a muscle can produce and it has been proven that they can be measured using conventional transverse ultrasound scans [1,2,3]. In addition, echogenicity is also important for detecting health disorders affecting the muscles; in certain diseases, intramuscular fat and fibrosis alter normal muscle composition causing a progressive increase in echogenicity [4]. However, manually placing the region of interest (ROI) in each muscle to extract the echogenicity can be tedious, user-dependent, and time-consuming. Therefore, the automatic extraction of CSA via state-of-the-art deep learning algorithms is an important research field that has witnessed a growing interest in recent years. Its goal is to quantify the muscle size and echogenicity inside the muscle CSA since it can be experimentally determined that a maximum ROI size within a given muscle will produce the least measurement variation.

A few methods have been used to estimate muscle CSA in vivo. These include magnetic resonance imaging (MRI), computed tomography, and ultrasound imaging. It has been shown that ultrasound imaging produces reliable results compared to MRI, which is considered the golden standard [5]. The extraction of the muscle’s anatomical cross-section area (ACSA) is commonly performed by positioning the transducer in the transversal plane of the muscle (i.e., across the largest muscle diameter) and using an extended field-of-view imaging technique which makes the recording of the larger portion of the muscle possible. Although this imaging modality contains more information about muscle characteristics, the acquisition process is complex. It requires high experience on the operator’s part, high accuracy of manual tracking, dedicated software tools, and is time-consuming. A simpler and more accurate way of performing this measurement is by extracting the visible muscle CSA using conventional transverse ultrasound scans. Specifically, in [6,7,8], the authors evaluated the muscle size changes induced by musculoskeletal training and rehabilitation by analysing the visible muscle CSA. Furthermore, in other studies [9,10], it has been reported that a CSA variation >5% is an indicator of a clinically relevant change; therefore, the accurate extraction of this measurement is important.

However, delineating the CSA from the transverse MSK-US scans is a time-consuming and user-dependent task, thus making it error-prone. Therefore, recent studies have focused on developing automated ways to extract the CSA without manual intervention using image processing and deep learning techniques to address this issue. In particular, in [11,12], the authors introduced a gradient-based filtering approach to initially delineate the aponeurosis-like structures of the examined images and later used heuristic and morphological operations to identify the relevant deep and superficial aponeuroses. As a final step, the CSA area was obtained by connecting the endpoints of the aponeurosis. However, their approaches’ downsides are that the aponeuroses’ identification is based exclusively on gradient-based filtering, which can be error-prone in low-contrast images, a common situation in MSK-US scans of abnormal muscles.

To overcome this difficulty, in [13] the authors investigated the performance of different deep learning architectures in automatically delineating the CSA in transverse MSK-US images in a relatively large-scale dataset of three different muscles. Additionally, they used the z-score of the CSA echogenicity to classify the muscles as normal and ab-normal based on reference values from healthy subjects. Their results indicate that it is feasible to automatically segment and perform quantitative grayscale analysis in the CSA with comparable performance to human experts. However, as the authors state, their study’s limitations are the small number of the examined skeletal muscles and the fact that all of their recordings were taken using a specific ultrasound machine. Similarly, in [14], the authors proposed an automatic tracking method for the cross-sectional muscle area in ultrasound images of rectus femoris using a convolutional neural network during muscle contraction. Different approaches have also been used in analysing MSK-US images for the automatic extraction of ACSA. In particular, in [15], a semi-automatic ImageJ script (named “ACSAuto”) for quantifying the ACSA of lower limb muscles was proposed, and in [16], a UNet [17] was trained to automatically segment ACSA in panoramic ultrasound images of the human lower limb muscles.

Convolutional neural networks (CNNs) have not been used only for automating the CSA measurement. In recent studies, the effectiveness of deep learning methods has also been demonstrated in other medical image segmentation problems. In particular, in [18], a deep learning approach was used to automatically extract the muscle thickness, fascicle length, and pennation angle from MSK-US longitudinal scans of three different muscles. The authors initially employed Attention-UNet [19] to segment the deep and superficial aponeuroses, and then the measurements were extracted by incorporating a heuristic-based approach. Another notable recent development was the introduction of vision transformers [20,21], which attracted a growing interest in medical imaging for their ability to capture global context information compared to CNNs with local receptive fields. Specifically, in [22], TMUNet is presented, which is a UNet variant with transformer capabilities. The authors integrated a contextual attention mechanism to adaptively aggregate pixel, object, and image-level features. Furthermore, they coupled a transformer module with the CNN encoder to model an object-level interaction. In addition, in [23], a convolutional multilayer perceptron (MLP)-based network called UNeXt was proposed, which was evaluated in breast ultrasound image segmentation. Its advantage is that MLPs are less complicated than convolution or self-attention and transformers, so reducing the number of parameters and computational complexity was feasible.

Several studies have emerged in recent years regarding the textural analysis of musculoskeletal ultrasound images [24,25,26,27,28]. The purpose of these studies is usually to categorise subjects’ biomarkers based on muscle texture, such as gender, the dominant side, the body mass index (BMI), or the pathological condition of the muscle, especially in specific musculoskeletal disorders. The methods mostly used for the categorisation belong to classical machine learning with hand-crafted feature extraction techniques and classical classifiers. However, deep learning methodologies have recently been used to characterise muscle texture. In [27], the authors used three common deep learning architectures to classify a muscle region of interest (ROI) as sarcopenic based on its texture. In addition, the Grad-CAM [29] analysis was applied to visualise the exact location inside the ROI that played the most significant role in the classifier decision. An accuracy ranging from 70.0% to 80.0% for predicting sarcopenia was reported in their experiments, showing that such a diagnostic tool is feasible.

This study presents a complete pipeline for automatically extracting the muscle CSA and its mean grey level value, representing echogenicity. Different state-of-the-art deep learning models and vision transformers have been incorporated along with customised procedures to extract this information automatically. Subsequently, comparative muscle size and echogenicity results are reported and discussed for patients of different age groups and health conditions, as well as the behaviour of these measurements in different muscle sections. Additionally, a textural analysis is performed in the muscle CSA of these subjects to investigate whether a classification into healthy and abnormal muscles is feasible by using higher order and more sophisticated information than echogenicity. In particular, a deep learning classifier trained in the muscle CSA texture of young and elderly subjects is used to automatically classify the ultrasound recordings into these categories. Finally, the performance of each muscle section in this classification was investigated to find which muscle was more fitted for this task.

This study’s main contribution is the improvement of the existing methods for a more efficient and reliable calculation of the muscle CSA, by introducing state-of-the art techniques such as vision transformers. Furthermore, future integration of this software in ultrasound machines would be of high value since this would allow ultrasonographers to calculate muscle size and echogenicity faster, more accurately, and in a more standardised fashion. Another contribution is automatically classifying the subject’s muscle condition solely from the CSA texture, again with a state-of-the-art deep learning classifier. To the best of our knowledge, this is the first time a complete pipeline is presented, beginning from the automatic extraction of the CSA and ending in classifying an abnormal muscle. Based on our results, we firmly believe that the proposed method can be applied in the clinical routine as an additional diagnostic tool to assist the experts. Finally, it is worth mentioning that all of the analyses have been performed on a novel, large, and diverse MSK-US dataset; therefore, the results presented in this study provide additional knowledge for the feasibility of the abovementioned tasks.

## 2. Materials and Methods

### 2.1. Database

The ultrasound recordings used in this study were acquired from 210 subjects from May 2017 to November 2019 in the Rehabilitation Department of the University Hospital of Patras by the same experienced musculoskeletal sonographer (N.B.). All of the acquisitions were performed using a Logiq P9 ultrasound system (G.E. Healthcare GmbH, Freiburg, Germany) with an ML6-15 linear array transducer operating at 10-MHz. All image optimisation modes were disabled to eliminate modification of image characteristics by software processing, except for harmonic tissue imaging. The gain was set to 50 and the dynamic range to 66 dB, and both were kept constant throughout the examination of all subjects. The examination protocol of the recording sessions was fixed for the selected muscles and kept consistent for all of the participants. In particular:

- The transverse ultrasound scans of the tibialis anterior (T.A.) muscle were fixed at one quarter of the distance from the inferior pole of the patella to the malleolus lateralis.
- The transverse ultrasound scans of the rectus femoris (R.F.) muscle were fixed halfway along the line from the anterior–superior iliac spine to the superior pole of the patella.
- The transverse ultrasound scans were fixed at the bulkiest part of the medial head of the gastrocnemius (GCM) muscle.
- The transverse ultrasound scans of the anterior arm muscles were fixed at two thirds of the distance from the acromion to the elbow crease. This section included the biceps brachii and brachialis (B.B.) anterior muscle.

The imaging depth was set at 4 cm for all muscles except for the rectus femoris, which was set at 6 cm. This depth was increased in the case of patients with voluminous muscles to include the entire muscle in the image. The focal zones (up to six) were distributed evenly along the depth of the image. A generous quantity of ultrasound gel (CLEAR ECO Supergel, Ceracarta S.p.A., Forli, Italy) was used to achieve the best possible ultrasound beam penetration and to prevent deformation of the soft tissues attributable to transducer pressure during the examination. The transducer was kept perpendicular over the muscle fascia (i.e., the beam inclination was adjusted to achieve the brightest echo from the muscle fascia) to obtain the images in a standard and uniform manner.

A total of 2005 ultrasound images of 4 different muscles from 210 subjects in different age groups and health conditions were analysed. In particular, 116 of the participants were young (mean age of 25.3 ± 4.9) and healthy, 76 of them were elderly (mean age of 75.3 ± 6.6) and non-sarcopenic, and, finally, 16 were elderly (mean age of 77.3 ± 6.4) and sarcopenic [30]. The total number of ultrasound examinations of the participants was 253 since a small number of the young and healthy participants were examined multiple times in a short period for the study presented in [31]. After visual inspection of the U.S. images of these secondary examinations, it was decided to include them in the study since their muscles’ visual and geometrical characteristics were distinct at each examination; therefore, the contamination risk of the dataset was minor. Parts of this dataset have also been published in [18,25], but the objectives of these studies were different from the current one. Therefore, this is the first time the complete dataset has been analysed for the automatic extraction of the CSA and its corresponding mean grey level value, representing the muscle’s echogenicity.

For simplicity, we divide the dataset into three groups with common characteristics. Group 1 consists of measurements of the young and healthy individuals, Group 2 of the elderly and non-sarcopenic subjects, and Group 3 of the elderly sarcopenic patients. It must be noted that the diagnostic criteria followed in this study for a patient to be considered as sarcopenic were proposed in the European Working Group on Sarcopenia in Older People (EWGSOP2) in the revised European consensus. Additional information regarding the ultrasound examination procedure and the measurements of Groups 2 and 3 can be found in [30]. Finally, in Table 1, the demographic characteristics of each group are presented.

**Figure 1:**
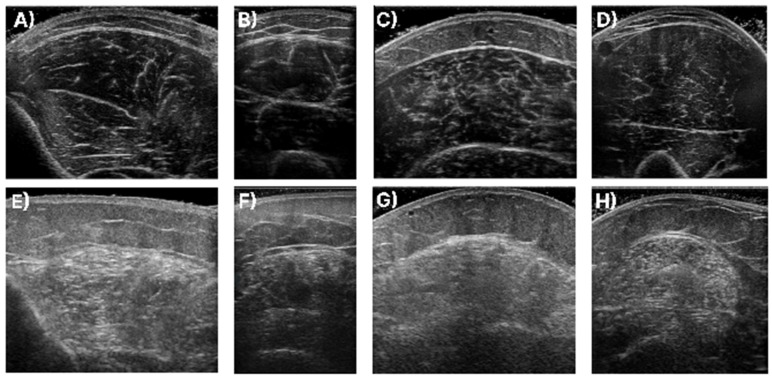
Sample of ultrasound recordings with normal and high echogenicity. (**A**,**E**) shows images from the T.A., (**B**,**F**) shows images from the R.F., (**C**,**G**) shows images extracted from the GCM, and finally, (**D**,**H**) are images of the B.B.

In Figure 1, samples of the studied muscles are presented for normal and high echogenicity images. It is observable in these images that the muscle aponeuroses, especially in the high echogenicity images, are hard to distinguish from the other muscle tissue, making the accurate localisation of the CSA more challenging.

### 2.2. Database Annotation

**Figure 2:**
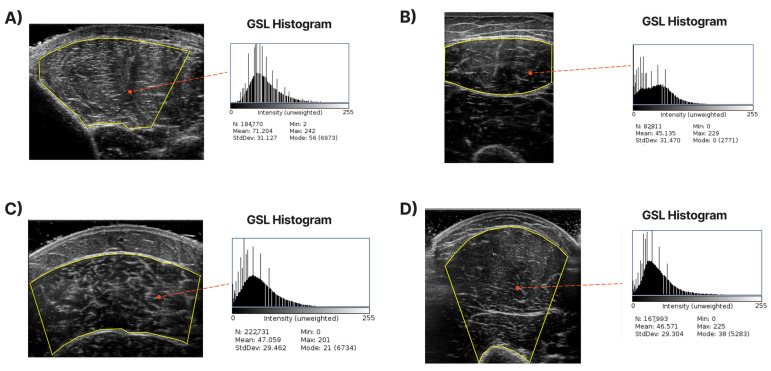
Sample of ultrasound images with their corresponding annotation. The CSA area contours are depicted with yellow lines. The grayscale level (GSL) histogram and its mean value are extracted inside the CSA. (**A**) shows the measurements of T.A., (**B**) shows the measurements of R.F., (**C**) shows the measurements of the GCM, and (**D**) demonstrates B.B.

An accurate definition of the CSA in each examined muscle is required to develop a system to extract it automatically. For each muscle, the expert has annotated the maximum visible cross-sectional area (highlighted with the yellow contour in the images of Figure 2) based on his experience but also following the guidelines presented in [4,11]. Specifically, for the T.A., R.F., and B.B., the transverse recording was sufficient to capture the entire muscle in most cases. For the GCM, the whole visible part of the muscle was labelled as the CSA. We tried to omit the areas near edges where artefacts existed since they do not accurately represent the inner muscle and can cause deterioration in the accuracy of muscle echogenicity measurements. In Figure 2, some images, along with their annotated counterparts, are presented. Finally, from the pixels inside the CSA, the mean grayscale level value was calculated to represent the muscle echogenicity as a singular value. This mean (µ) was calculated from the following formula:

(1) $$µ=\frac{\sum_{i=1}^{N}{µ}_{i}}{N}$$

where ${µ}_{i}$ is the grayscale value of the *i*th pixel, and *N* is the total number of pixels of the cross-sectional area. Since the CSA was defined in the image plane, the last step was to transform the pixels into physical units (mm^2^) by multiplying accordingly by the pixel size retrieved from the DICOM metadata provided by the ultrasound machine.

**Figure 3:**
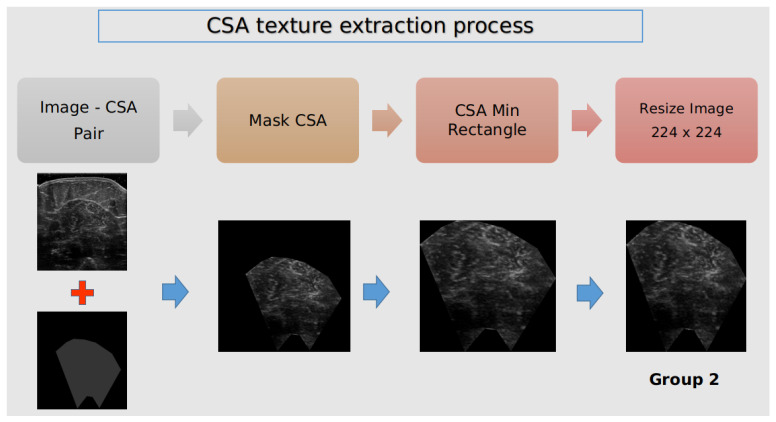
The process of extracting and isolating the CSA texture at each ultrasound recording.

After the CSA annotation, the texture of the cross-sectional area from the examined ultrasound recordings was isolated for further analysis. Our goal was to investigate whether we could distinguish which group the muscle belongs to solely from the image texture. Figure 3 presents the pipeline for CNN’s input image construction.

1. The original image was masked from the annotated CSA to remove irrelevant information and noise affecting the classifier’s performance.
2. The minimum area rectangle of the CSA binary mask was extracted to remove some padding and only keep the desired ROI.
3. As input to the classifier, all of the newly created images were resized to 224 × 224 and labelled to the corresponding group.

### 2.3. CSA Extraction

#### 2.3.1. Introduction

**Figure 4:**
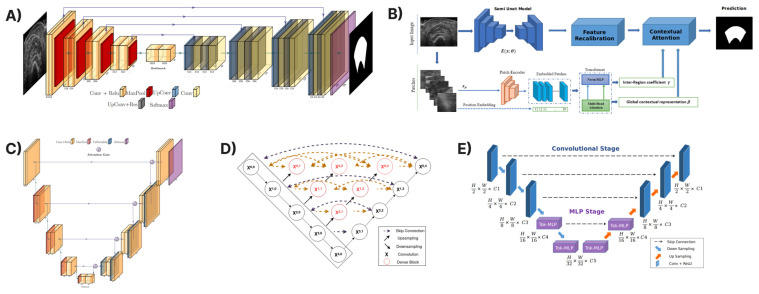
The deep learning architectures used in this study to automatically segment the CSA. In (**A**), the original UNet [17] is depicted; in (**B**), the vision transformer TMUNet [22] is depicted; in (**C**), the Attention-UNet [19] model is depicted; in (**D**), the UNet++ [32] is depicted; and finally, in (**E**), the UNeXt [23] architecture is depicted.

In this section, several state-of-the-art deep learning models which have been used for the automatic delineation of the cross-sectional area are presented. These convolutional neural networks have been successfully applied to different medical problems in the recent past. In particular, UNet [17], Attention-UNet [19], UNet++ [32], UNeXt [23], and TMUNet [22] have been included for assessment in the examined dataset of the transverse ultrasound images of the four muscles. Figure 4 presents a visual representation of the deep learning architectures.

#### 2.3.2. UNet

The UNet architecture, which has been used successfully in numerous problems in medical segmentation in recent years, consists of a contractive path that enables it to capture context and an expansive path that enables precise localisation. In the contractive path, 3 × 3 convolutions are applied, each followed by a rectified linear unit (ReLU) activation and a 2 × 2 max pooling operation for downsampling. At each downsampling step, the number of feature channels is doubled. In the expensive path, an upsampling of the feature map is applied, followed by a 2 × 2 convolution that halves the number of feature channels, a concatenation with the correspondingly cropped feature map from the contracting path, and two 3 × 3 convolutions, each followed by a ReLU. Finally, a 1 × 1 convolution maps each 64-component feature vector to the desired number of classes at the final layer.

#### 2.3.3. Attention-UNet

Attention-UNet is another state-of-the-art deep learning architecture used in this study. It is an adaptation of the original UNet architecture, with attention gates (AGs) added to the skip connections to emphasize better salient features during the training procedure. The authors claim that these attention gates can filter irrelevant and noisy responses in the convolutional network skip connections that could otherwise result in false-positive predictions for small objects with significant shape variability. To integrate just relevant activations, the aforementioned is conducted prior to the concatenation procedure. Another important fact is that both during the forward and the reverse pass, AGs filter neuron activations. Gradients originating from background regions are downweighted during the backward pass, allowing model parameters in shallower layers to be updated, based mostly on spatial areas relevant to a given task.

#### 2.3.4. UNet++

The UNet++ design is a heavily supervised encoder–decoder network, where many dense, nested skip connections are connected to the encoder and decoder sub-networks. The semantic gap between the feature maps of the encoder and decoder sub-networks is intended to be closed by the newly developed skip paths. The underlying premise of the architecture is that when high-resolution feature maps from the encoder network are gradually enriched before fusion with the corresponding semantically rich feature maps from the decoder network, the model can efficiently capture fine-grained details of the foreground objects. Furthermore, according to the authors, the hypothesis is that the optimiser would face an easier optimisation problem if the received encoder feature maps and the corresponding decoder feature maps were semantically similar.

#### 2.3.5. UNetXt

The UNeXt still follows the UNet’s 5-layer-deep encoder–decoder architecture with skip connections, but each block’s design is different. In particular, UNeXt has a convolutional stage with fewer filters in the network’s start and end blocks, followed by an MLP stage. A unique tokenized MLP was proposed, which is effective at maintaining a reduced computation burden since it considerably reduces the number of parameters while preserving performance in the network bottleneck. In particular, the encoder processes the input image through 3 convolutional blocks and 2 tokenized MLP blocks. Later, two tokenized MLP blocks and three convolutional blocks make up the decoder. Every decoder block raises the feature resolution by 2 while every encoder block lowers it by 2.

#### 2.3.6. TMUNet

The contextual attention network (TMUNet) offers a two-stream pipeline, wherein the first stream employs a CNN module to extract local semantic information and the object-level interaction map. The second path incorporates the transformer module to capture long-range contextual representations. The transformer module produces an image-level contextual representation (ICR) to construct the spatial dependency map at the image level. It also produces regional importance coefficients (RIC) to model the importance of each region. In addition, this method includes a contextual attention module to scale the feature maps adaptively and emphasises the important regions. Empirical findings validate that this method can pay more attention to the overlapped boundary area while providing a strong semantic segmentation map.

#### 2.3.7. Post-Processing

**Figure 5:**
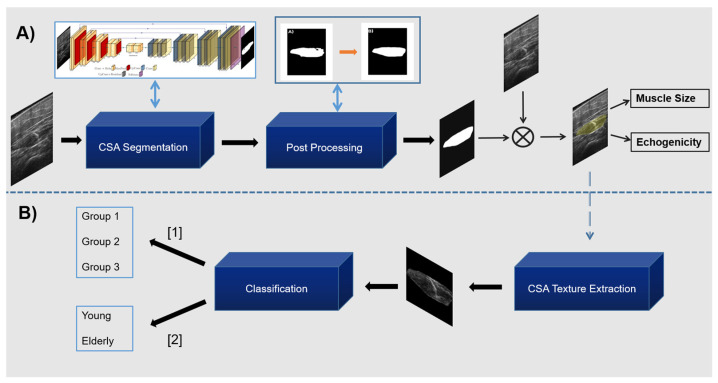
In (**A**), the flowchart of the proposed pipeline to extract the CSA and its mean grey level (echogenicity) is presented. In (**B**), the two classification analyses from the predicted CSA texture are depicted.

Since the results of the CNNs often include artefacts and noise, a post-processing refinement was induced to improve the predicted segmentation maps. The artefacts were associated mainly with three cases. In the first case, there were false detections of small areas outside of the muscle CSA. In the second case, there were gaps and irregular points inside the mask. Finally, in the 3rd case, there were irregular shapes of the boundaries of the mask that needed to be improved. The segmented mask contour was extracted initially to address these irregularities, and the one with the largest area in terms of pixels was kept. Subsequently, morphological operations were performed in this mask to fill possible gaps inside it and to smoothen any sharp edges in the boundaries. In Figure 5A, the proposed pipeline for the CSA delineation is demonstrated, ending in the calculation of the medical measurements.

#### 2.3.8. Evaluation Protocol and Training Strategy

**Figure 6:**
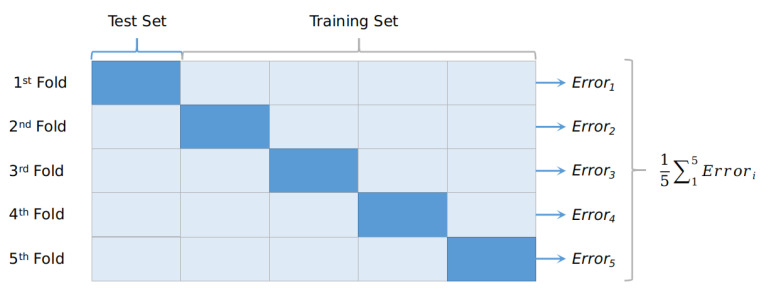
The 5-fold evaluation protocol. At each iteration, 80% of the examinations were considered training sets and the remaining 20% were considered test sets. The average accuracy of all of the iterations was regarded as the model performance.

As an evaluation protocol, the k-fold cross-validation with k = 5, depicted in Figure 6, was selected since it ensures that every observation from the original dataset has the chance of appearing in the training and test set. This protocol split the dataset into k equal parts. The training process was repeated k-times; each time, k-1 parts consisted of the training set and the validation. The final performance was the average of all of the repetitions. Since our dataset consists of a few thousand images and not millions, it was computationally feasible to apply this in our task. This validation protocol is known for its ability to flag problems such as overfitting and selection bias and to give an insight into how the model will generalise to an independent dataset. In summary, cross-validation combines fitness measures in prediction to derive a more accurate estimation of model prediction performance.

All of the above-mentioned deep learning models except the TMUNet were trained similarly. For the TMUNet, we followed the training procedure described in the original paper [22] since it combines multiple objectives in its loss function and has a more sophisticated optimisation process. However, the input size of the network and the data augmentation techniques were the same as in the other architectures. In particular, the input of all the networks was resized to be 256 × 256 with batch size 8 and several epochs of 300. The selected loss function was the *Dice Loss* (2), where p is the prediction mask, g is the ground truth mask, and *N* is the total number of training samples.

(2) $$Dice\;Loss=1-\frac{2\sum_{i=1}^{N}p_{i}g_{i}}{\sum_{i=1}^{N}p_{i}+\sum_{i=1}^{N}g_{i}}$$

Finally, the ADAM optimiser was used for the optimisation process. Regarding the learning rate policy, a stepwise decrease in the learning rate was the best fitting. All the images during training were augmented using the following operations: vertical/horizontal flip, scaling, rotation, and sharpening.

### 2.4. CSA Texture Analysis

Following the successful CSA extraction using the above-mentioned deep learning architectures, two textural analyses in the muscle CSA were performed to examine:

- Whether there was any distinction in the muscle texture of the subjects of Group 1 (young and healthy), Group 2 (elderly and non-sarcopenic), and Group 3 (elderly and sarcopenic).
- Whether there was any distinction in the muscle texture of the young subjects (Group 1) and the elderly subjects (Group 2 and Group 3 combined).

In Figure 5B, a visual representation of these two experiments exists. In particular, from the predicted CSA and the original image, we isolated the muscle texture (this procedure is also depicted in Figure 3) and provided it as an input to a deep learning classifier. Then, the classifier categorised the muscle texture according to the analysis we ran. In the first analysis, the classifier predicted the Group that the image belonged to and in the second analysis it predicted whether the muscle texture belonged to a young or elderly subject. These analyses will be useful for revealing whether there are significant differences in the muscle CSA texture that can be exploited for developing diagnostic tools to recognise diseased muscles solely from muscle texture.

**Figure 7:**
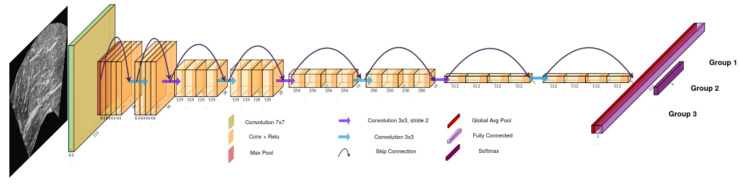
The Resnet18 classifier. For each image input, the classifier predicts which group the texture belongs to.

The incorporated deep learning classification model depicted in Figure 7 belongs to the well-established ResNet [33] family of deep learning architectures and is a lightweight version with 18 convolutional layers as the backend (ResNet18). Furthermore, regarding the experimentation setup, the preparation of each ultrasound image is shown in Figure 3. Finally, as before, the same 5-fold evaluation protocol was used to assess the classification accuracy of the decision model.

### 2.5. Evaluation Metrics

Several evaluation metrics were used to assess the CSA extraction performance. First, regarding the image segmentation problem, five well-established indexes were incorporated [34]. First was the dice coefficient (DSC) index, which measured the pixel overlap between two sets of data, and second (similarly to the DSC) was the intersection over union (IoU) index. Furthermore, precision and recall of the segmentation maps were also reported between the manual and automatic measurements, as well as the Hausdorff distance (HD95) at the 95th percentile, which computed the highest distance between the two segmentation maps.

Additionally, the mean discrepancy between the measurements was calculated regarding the root mean square error (RMSE) in physical units (mm^2^). Furthermore, the intra-class correlation coefficient (ICC), type ICC (2, 1), and Pearson correlation coefficient were used to evaluate the results statistically. Moreover, Bland–Altman analysis was incorporated to evaluate possible bias and systematic error in the manual vs. automatic CSA measurements. Finally, regarding the CSA texture classification problem, the precision, recall, and F1-scores were calculated to evaluate the classifier’s performance along with the corresponding confusion matrix of each analysis.

## 3. Results

### 3.1. CSA Extraction Results

Table 2 demonstrates the image segmentation results for the five examined network topologies and TRAMA algorithm. It must be noted that the implementation of the TRAMA algorithm is ours since there is no official one. However, we tried to follow as closely as possible the algorithm proposed in [11]. For all of the networks, the average performance between the evaluation set of the five folds is reported. In all five metrics, the TMUNet exhibits superior performance in relevance with the other networks, and therefore all of the CSA segmentation results needed for the rest of the analyses of this study were extracted using it. In particular, the best-reported precision is 0.95, with the corresponding recall being 0.96 showing the network’s capability to localise the deep and superficial aponeuroses accurately. Furthermore, in terms of DSC and IoU, the reported results are equal to 0.96 and 0.92, respectively, with the corresponding accuracy in HD95 being only 10.09, another indicator of the excellent performance of the segmented masks of the validation set. Lastly, the small standard deviation of the results indicate that the performance of the deep learning models is relatively stable without severe or total failures.

It must be noted that all of the different deep learning architectures surpassed the TRAMA methodology by a large margin. This behaviour can be explained by the convolutional neural networks’ ability to filter the noise better than the traditional approaches. More specifically, from the visual inspection of the results, TRAMA performed better in the recordings belonging to the young and healthy subjects (Group 1) since, in these images, the aponeuroses are more distinct and have higher contrast to the muscle tissue in comparison with those of the elderly (Group 2 and Group 3). In particular, the average precision of the TRAMA algorithm in all of the samples equals 43%, which is significantly less than that of CNNs. Additionally, the high standard deviation of the evaluation metrics highlights that the TRAMA predictions are not stable and that there are a lot of cases with significant or even total failures. This is the reason we omitted to report the HD95 metric in this experiment, since due to these failures becomes invalid.

Continuing our analysis, Table 3 compares the proposed method, which consists of the TMUNet and the post-processing refinement, to the manual measurements provided by a human operator for calculating the CSA size and their corresponding echogenicity in physical units. It presents the mean ± standard deviations and their discrepancy in RMSE and HD95, and their reliability as measurements in terms of the ICC metric and Pearson coefficient.

**Figure 8:**
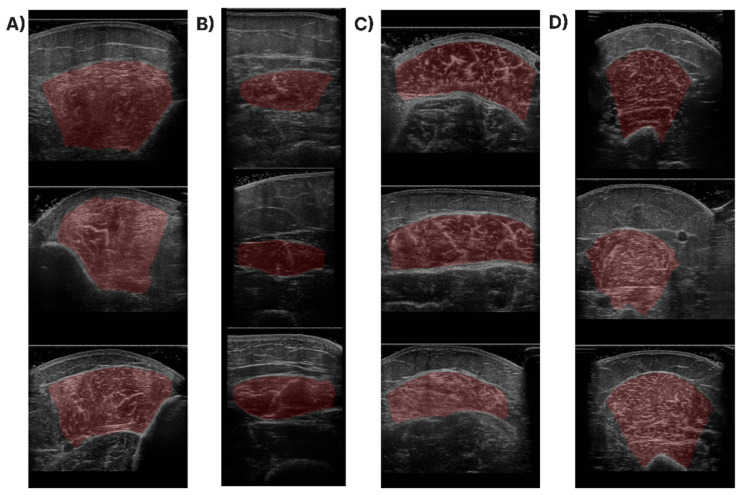
Qualitative results of the CSA segmentation for samples of the (**A**) T.A., (**B**) R.F., (**C**) GCM, and (**D**) B.B. The red areas are the predicted masks superimposed onto the input image.

From the results in Table 3, it can be noted that manual vs. automatic measurements have an extremely low RMSE and HD95, showing the high quality of the predicted cross-sectional area. In particular, the RMSE equals 38.15 mm^2^ which equals 4% of the total CSA, a significant result that indicates that the proposed method can be used for future automation of this clinical task. Regarding the HD95, the average performance equals 2.20 mm, which indicates the high quality of the produced masks. Regarding the statistical analysis of our results, the ICC and Pearson coefficient are close to 1, showing the reliability of the two measurements. Furthermore, in terms of echogenicity, the RMSE difference between the two readings is only 0.88, a discrepancy near 1% that shows that these two measurements can be used interchangeably. Finally, in Figure 8, qualitative results of the proposed method are provided in different samples of each examined muscle section. From the qualitative results of Figure 8, it is clear that the predicted masks after the post-processing refinement are very accurate and managed in most cases to capture the entire muscle CSA in all of the different muscle sections.

**Figure 9:**
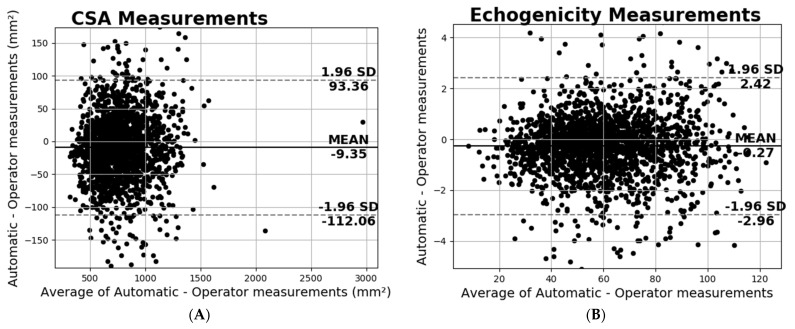
Bland−Altman plots of the (**A**) CSA and (**B**) echogenicity in all the databases.

Another useful analysis presented in Figure 9 is the Bland–Altman plot of the CSA and echogenicity measurements. Regarding the CSA measurements in Figure 9A, the plot shows negligible additive bias and that most differences fall between the 95% limits of agreement. Finally, there are no distinguishable patterns depicted in the plot. Considering the echogenicity measurements in Figure 9B, once again in this Bland–Altman plot, there are no distinguishable patterns and neither systematic error is presented.

Continuing our analysis, Table 4 compares the automatic vs. the manual CSA measurements and their corresponding echogenicity in all of the examined muscles. For each muscle, the average discrepancy is presented in terms of RMSE.

From the above results, the B.B. has the largest CSA in all of the examined muscles in both readings. This result is expected since the B.B. consists of two muscles (biceps brachii and brachialis anterior) commonly functionally considered as one unit. Regarding the average discrepancy between the two readings, R.F. has the lowest RMSE, equal to only 32.86 mm^2^. In terms of percentage, the B.B. and GCM have a 5% difference between the manual and automatic measurements, the TA has a 6% difference, and the best result was reported in R.F. with only a 4% difference between the two readings. Regarding the echogenicity, R.F. again shows the lowest absolute value at 44.78, and T.A. presents the largest at 70.48. Finally, regarding the average differences, B.B. demonstrates the best result, equalling only 0.73, with all the other muscles following nearby.

**Figure 10:**
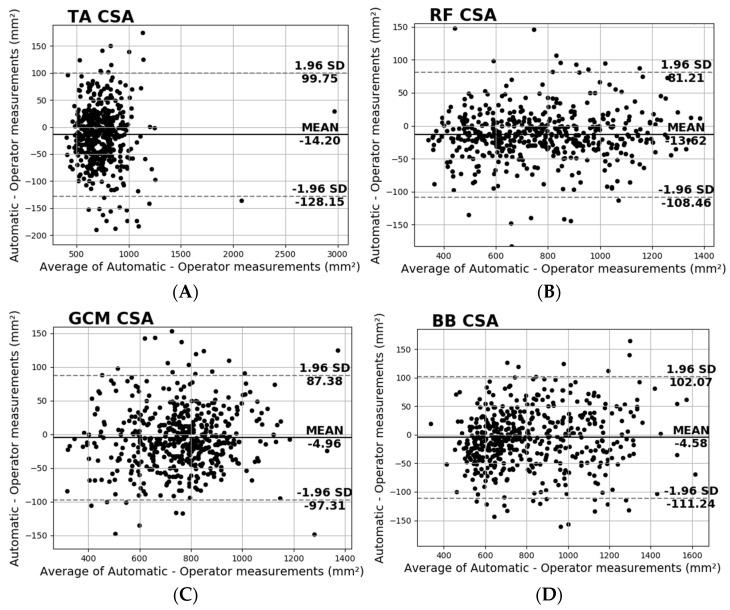
Bland−Altman plots of the CSA for the (**A**) T.A., (**B**) R.F., (**C**) GCM, and (**D**) B.B.

Additionally, in Figure 10, the Bland–Altman plots depict the performance of the four examined muscles. These plots show negligible additive bias and no systematic errors since most differences fall between the 95% limits of agreement. Finally, there are no distinguishable patterns depicted in the plots.

Furthermore, Table 5 presents an analysis of the three groups that participated in this study, defined in Table 1. Specifically, the CSA and its corresponding echogenicity are reported along with its average discrepancy in RMSE.

From the results above, it is clear that Group 1, consisting of young and healthy subjects, has the largest muscle cross-sectional area and the lowest echogenicity. This result is expected since the young subjects are anticipated to have the best muscle architecture and characteristics. In particular, the average automatic CSA measurement between all of the recordings was found to be 841.06 mm^2^, differing by only 34.90 in terms of RMSE from the manual readings. The corresponding average echogenicity was reported to be 52.42, once more very close to the manual measurements. Additionally, the CSA measurements of Group 2 and Group 3 were nearby in average magnitude. Therefore, this can be explained by the fact that both groups include the elderly population. The difference between them is that the subjects of Group 3 are sarcopenic; hence, the lower CSA and the larger echogenicity reported in this group’s measurements are compatible with the clinical diagnosis.

**Figure 11:**
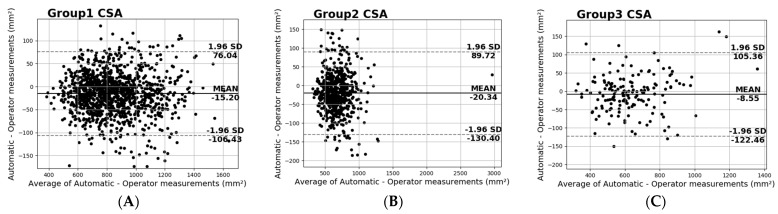
Bland−Altman plots of the CSA for (**A**) Group 1, (**B**) Group 2, and (**C**) Group 3.

Another useful analysis presented in Figure 11 is the Bland–Altman plots of the CSA measurements per study group. It is clear from the plots that most of the samples belong to the Group 1 category, and the smallest number of the samples are in Group 3. Furthermore, the plots show negligible additive bias and no systematic errors since most differences fall between the 95% limits of agreement. Lastly, once more there are no distinguishable patterns depicted in the plots.

**Figure 12:**
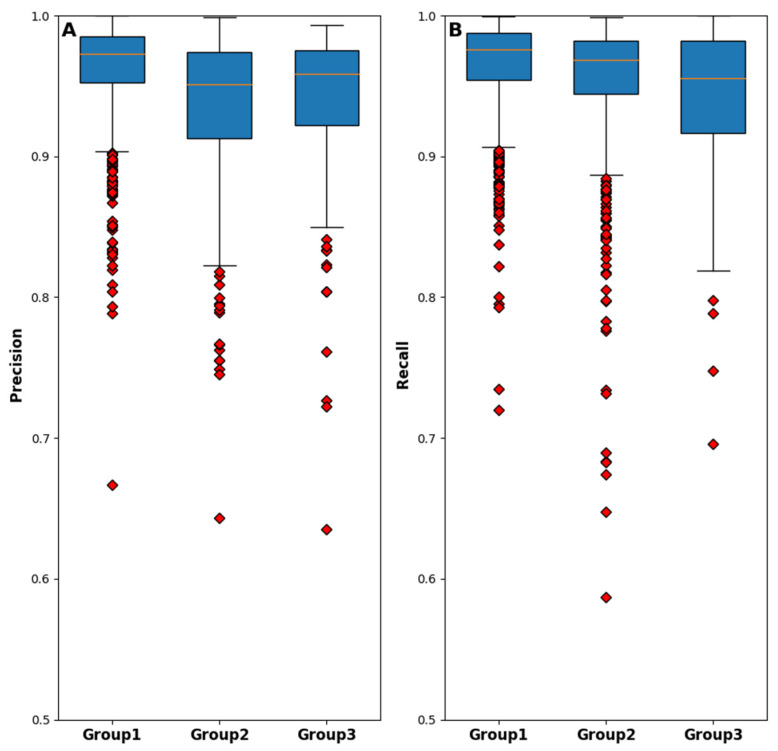
Box plot diagrams of the automatic method performances in terms of (**A**) precision and (**B**) recall metrics, dividing the dataset between the groups.

Finally, for assessing the normality of the precision and recall distributions for each examined group, the box plot diagram is presented in Figure 12. It is observable from this diagram that the distributions failed to match a normal distribution since skew is presented in all of them. Furthermore, it must be noted that the box plots of Group 1 are comparatively shorter than those of the other groups, indicating that these measurements have a higher level of agreement with each other.

### 3.2. CSA Textural Analysis Results

This section presents the textural analysis of each group’s CSA. Table 6 presents the classification results for the manually and automatically extracted CSA image textures. Each method’s performance is reported in terms of the classification metrics described in Section 2.5.

**Figure 13:**
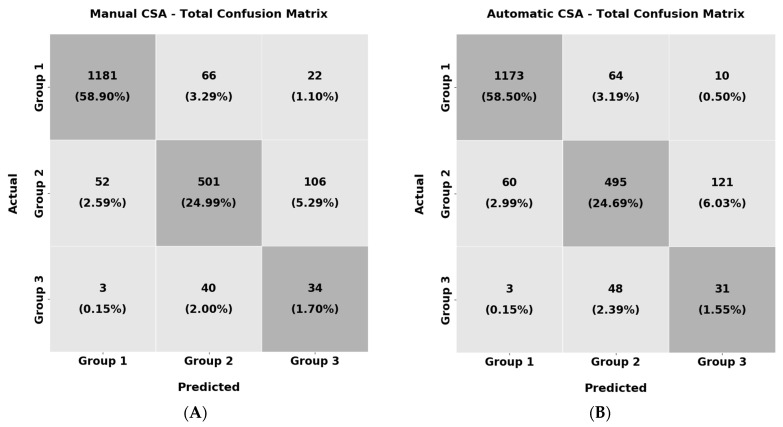
Confusion matrices for the per-group analysis. In (**A**), the manually extracted CSA textures were used and in (**B**), the automatic were used.

From the results above, it can be highlighted that the classifier labelled most of the samples correctly, over 84% of the time. Furthermore, the manually extracted CSA and the image segmentation network predictions have almost identical performance in the three group categories’ classification problems. Although, since the dataset suffers from imbalance, apart from the weighted average performance depicted in Table 6, the confusion matrices are also extracted in Figure 13 for analysing the per-group accuracy.

From the results in Figure 13, the texture of the muscle CSAs of Group 1 is more distinguishable than the other two groups. In particular, the images of this group achieve more than 93.1% accuracy in this task. The images of Group 2 also performed well, with an average precision of 78.4%. In contrast, we observe that the results of Group 3 deteriorate to nearly 44.2% and it is common for the classifier to mislabel them as Group 2 and vice versa. The above can be explained by the fact that in both Group 2 and Group 3, many muscle CSAs have high echogenicity and similar textural characteristics that cause this confusion in the classifier. Additionally, the same pattern in the results is observed in the automatically extracted CSAs. In the textural analysis of the predicted CSAs, the classification task in Group 1 achieved 94.0% accuracy, followed by Group 2 with 73.2% and Group 3 with 37.8%. Once more, the same confusion in the classifier occurs between the samples of Group 3 and Group 2.

**Figure 14:**
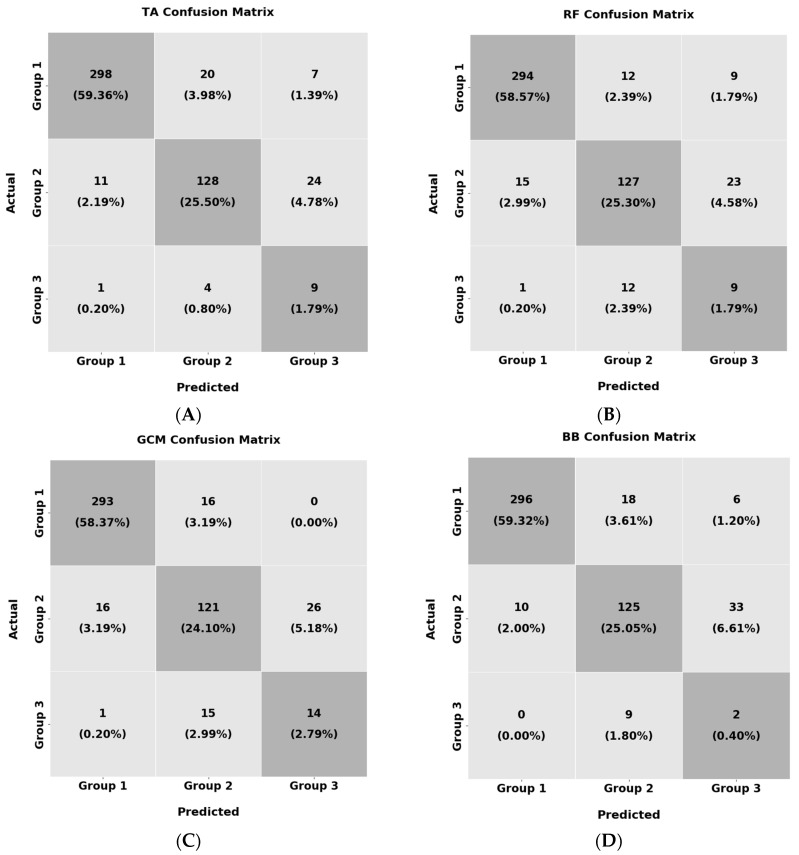
Confusion matrices for the group classification for the (**A**) T.A., (**B**) R.F., (**C**) GCM, and (**D**) B.B.

Furthermore, it must be noted that the number of individuals in Group 3 is much less than in the other two categories, an important limitation to consider. Additionally, even though the patients of Group 3 are sarcopenic, not all muscles are equally affected. Therefore, it is possible that in several muscles, the effects of this disease are more severe than others, as already presented in [30], which can lead to a performance reduction. Finally, some of the muscle sections of the elderly subjects in Group 2 can suffer from other pathological conditions, such as obesity, since sarcopenia is not the only condition than can cause deterioration in muscle architecture. Figure 14 presents the confusion matrices for each examined muscle for the manually extracted CSA.

From the results in Figure 14, it must be noted that all of the examined muscles showed similar performance. Specifically, the average accuracy of the T.A. was 86%, that of the R.F. was 84%, that of the GCM was 85%, and finally that of the B.B. was 81%. In all muscles, the most significant accuracy reduction was due to confusion of the classifier in the samples of Group 3 as Group 2 and vice versa. Lastly, it is worth mentioning that the GCM presented the best results concerning the classification accuracy of Group 3 which indicates that this muscle is more suitable for distinguishing sarcopenic patients from healthy individuals.

**Figure 15:**
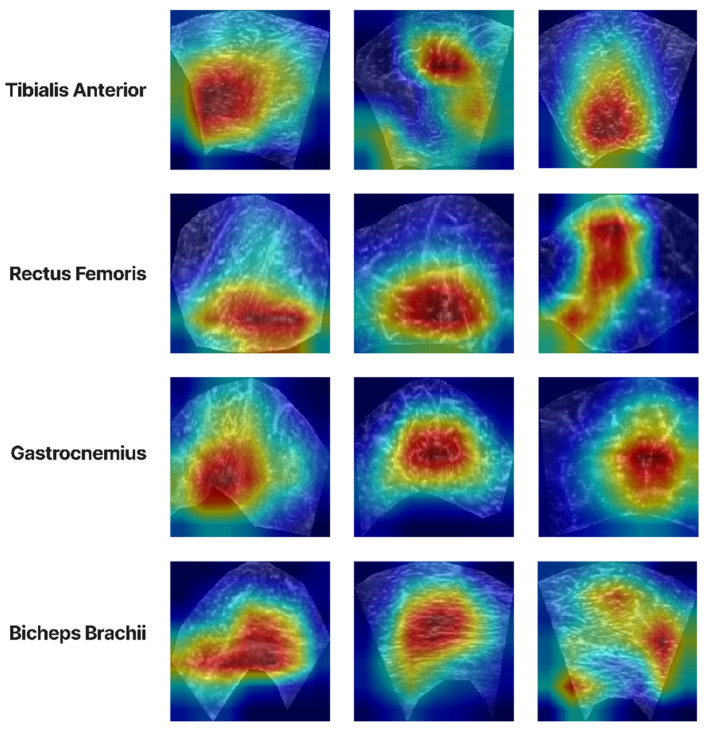
Grad-CAM analysis for the group classification of each muscle section.

Finally, the Grad-Cam [29] analysis was performed to investigate which areas of the activation maps were triggered when the classifier made a decision. This analysis will help us to interpret the results better and avoid any possible bias in the decision model. In Figure 15, the Grad-Cam results are presented. From these, it can be observed that the areas that have the most important role in the final decision are mostly texture intensive since they contain a lot of muscle tissue, indicating that the classifier concentrates on the CSA texture. Hence, this is an important result for creating a diagnostic tool that uses the image texture to make decisions about the pathological state of the muscle. However, it is also possible that the GCM and the B.B. shape of the CSA are other factors that affect the classification performance. In some cases, this can be derived from the fact that the most intense (red) activation is near an irregular contour of the CSA and not in a more central area. Further experimentation will provide better insights into the true capabilities of such a system.

**Figure 16:**
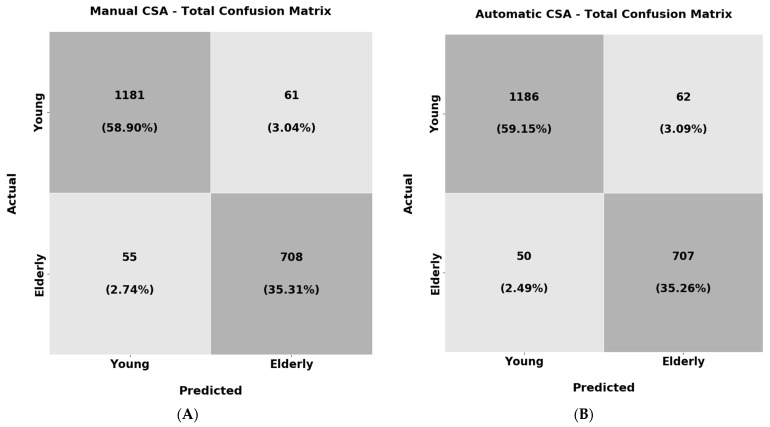
Confusion matrices for the young vs. elderly subjects’ analysis. In (**A**), the manually extracted CSA texture was used and in (**B**), the automatic was used.

In the last experiment of the texture analysis, the objective was to train a ResNet18 classifier to recognise the young individuals (Group 1) and the elderly subjects (Group 2 and Group 3 combined) solely from the muscle CSA texture. As before, the classification results for the manually extracted and automatic CSA are reported in Table 7, and their confusion matrices are shown in Figure 16.

From the results above, it must be highlighted that the classifier trained both in the manually extracted CSA textures as well as the automatic correctly labelled the majority of the samples over 94% of the time. Furthermore, the recall and F1 scores also present very high performance (94%) in both of the experiments. These results are an additional indicator that the muscle CSA texture can be used to distinguish the young subjects from the elderly ones. This could lead to diagnostic tools solely from the muscle texture that could be useful in clinical practice. Additionally, apart from the weighted average performance depicted in Table 7, the confusion matrices are also presented in Figure 16 for analysing the per-class accuracy.

From the results above, it is observable that the classifier shows almost identical performance in both experiments. Regarding the per-class accuracy for the manually extracted CSAs texture, the classifier reached 95.0% in the samples belonging to the young population and 92.7% for the elderly. For the texture of the automatically extracted CSAs, the corresponding precisions were 95.0% for the samples belonging to the young category and 93.3% for the elderly. One conclusion that can be made is that the young subjects again have more distinguishable muscle texture in comparison with the elderly subjects. Nevertheless, in the elderly subjects, the classifier could still recognise the vast majority of the samples correctly.

## 4. Discussion

This study employed different state-of-the-art deep learning architectures and vision transformers to segment the cross-sectional area in a new, large, and diverse musculoskeletal ultrasound database. After delineating the CSA, the muscle echogenicity was calculated using this area’s mean grey level intensity. The presented preliminary results regarding four very informative muscles for investigating neuromuscular disorders and sarcopenia [13,30], indicate that such an automated approach can be used successfully in clinical practice. Additionally, in a supplementary analysis, the texture of the muscle CSA (extracted manually and automatically) was investigated for its ability to recognise the subject’s group. Our results indicate that for the younger population, the differences in muscle texture in comparison with the elderly subjects were significant and therefore the classifier was capable of correctly categorising the majority of the samples. Regarding the recognition results of the elderly non-sarcopenic and sarcopenic patients, the classifier presented significant confusion between these two categories leading to performance reduction. However, larger studies with more balanced datasets are required to assess the feasibility of this task conclusively.

A significant advancement of this study in comparison with other recent works presented in [11,12] is the use of deep learning models to address this problem. Filter-based approaches are hard to design, more prone to overfitting, and have difficulties in handling the noise in real-world conditions. In particular, we demonstrated that the TRAMA algorithm [11] severely underperforms compared to all of the examined convolutional neural networks. The reason for this is that in ultrasound images with abnormal echogenicity, the differentiation in the muscle aponeuroses from the inner muscle tissues is limited due to their low contrast. Hence, this leads to severe algorithm failure since muscle aponeuroses cannot be adequately localised, causing significant errors in CSA estimation and corresponding echogenicity.

Another similar study has been recently presented in [13]. In this study, the authors also used deep learning techniques to improve the performance of the existing automated methods. Our additional contribution in relevance to their work is, firstly, the introduction for the first time of vision transformer technology for the automatic segmentation of CSA, which is the most recent advancement in the deep learning field. Secondly, our analysis was performed in a new, large, and diverse database; therefore, the results presented here provide additional knowledge for fully automating the CSA measurement. Furthermore, this database consists of ultrasound recordings acquired with a highly reproducible examination protocol since minimal pressure was applied in order to not deform the inner muscle tissues, hence adding reliability to the measurements. This is not true for the datasets studied in [11,12,13], since it is clear from the images that a non-measurable pressure (highly user-dependent) was applied by the transducer during the examination. This variable degree of pressure can lead to muscle tissue deformation with subsequent changes in the thickness and echogenicity of the studied muscle, with eventual erroneous measurements. Finally, it must be noted that the k-fold evaluation protocol (k = 5) was followed which is more immune to overfitting and selection bias, leading to a better estimation of the final performance since it considers all of the available samples.

For the problem of the automatic CSA delineation, the best convolutional neural network was found to be TMUNet, a recently proposed visual transformer [22]. In particular, the automatic measurements achieved over 95% precision and recall compared with a human expert’s manual annotation of the CSA. The other examined architectures have achieved similar performance, another indicator of the capability of the deep learning models of providing an automated solution to this problem. Furthermore, the route mean square error of the two readings in all of the 2005 ultrasound transverse recordings was found to be 38.15 mm^2^, a minimal error that indicates the success of the proposed methodology. Regarding the discrepancy in the mean grey level value of CSA representing echogenicity, the manual and the predicted values are almost identical, with the average difference being only 0.88, hence near to 1% in percentage. Additionally, from the Bland–Altman plots in all automated measurements, it was demonstrated that no systematic error appeared, and most of the measurements fell within the limits of agreement. Finally, regarding the statistical analysis of the results, ICC (2,1) reached 0.97, showing the level of agreement between the two readings. Furthermore, the Pearson coefficient reached 0.99, demonstrating that these measurements are highly correlated.

Additionally, different supplementary analyses were performed in the newly annotated database to obtain significant insights into the sample distribution. Initially, the measurements of the ultrasound recordings of the different muscles were extracted and compared. From there, it was found that the B.B. possesses, on average, the largest CSA in the examined dataset and T.A. the smallest. Regarding muscle echogenicity, the smallest was observed in the R.F. and the largest was observed in the T.A. Additionally, the smallest error between the two readings was achieved in the R.F. This result can be explained by the muscle shape; in most cases, R.F. was composed of a smaller pixel area compared with the other examined muscles. Furthermore, the group analysis highlighted that the subjects who were elderly (Groups 2 and 3) had significantly less CSA and more echogenicity than their younger counterparts (Group 1). The above-mentioned is expected since ageing leads to a reduction in muscle mass and an increase in muscle fat which causes higher echogenicity. Furthermore, the lowest muscle CSA was found in elderly patients suffering from sarcopenia (Group 3), a disease that further deteriorates muscle architecture. Moreover, in this group, the highest echogenicity was also found, another indicator of muscle loss. Finally, in Group 1, the automatic measurements achieved the best result (young and healthy). The explanation for this is that the recordings in this group have more distinguishable boundaries between the aponeuroses and the inner tissue due to healthier muscles than the other study groups. Regarding the clinical application of the presented results, in [10] it was reported that a CSA variation >5% indicates a clinically relevant change and a variation above 10% can be a sign of muscle atrophy; therefore, the proposed method presented in this study which achieved an average 4% discrepancy between the manual and automatic measurements can be considered to be reliable for the future integration of this software in ultrasound machines.

Another important contribution of this study is the textural analysis of the CSA using a well-established deep learning model. This analysis consisted of two important experiments. In the second experiment, we investigated whether there are differences in the muscle texture of the young (Group 1) vs. the elderly population (Group 2 and Group 3 combined). The preliminary results indicate that the texture of the muscle CSA of the elderly is very distinguishable compared to that of the young population. In particular, the classifier achieved over 94% overall accuracy between the two populations, showing that a diagnostic tool that can predict the condition of the muscle solely from its texture is feasible. Regarding the first experiment of the textural analysis, in which we tried to classify the muscle texture in the three examined groups of this study, the results was not conclusive. Although the classifier achieved over 84% correct categorisation on average, from the confusion matrix of these results we demonstrated that much of the accuracy was due to the imbalance of the dataset. In particular, the classifier properly categorised the vast majority of the samples belonging to Group 1 and Group 2 (93.1% and 78.4%, respectively) but severely underperformed in the samples belonging to Group 3 (44.2%). We attribute this result mainly to three reasons. Firstly, there is a high dataset imbalance in the number of samples per category and the sex distribution. The number of samples in Group 3 is significantly less than in the other two groups; most samples come from female patients. Secondly, the muscles are not affected uniformly by sarcopenia since, as presented in [30], in some, the muscle loss is more severe than in others. Lastly, the subjects belonging to Group 2 are elderly, and their muscle architecture and quality are also affected negatively by their age. For these reasons, larger studies are required for more conclusive results about the feasibility of this task.

It is also worth mentioning that this study has some general limitations. First, a limitation is that the proposed method was assessed in only four muscles from over 200 that the human body possesses; hence, further studies with a larger population and with more muscle sections are required to confirm the accuracy of our results. Nevertheless, since the proposed method is scalable, we are confident that these challenges can be overcome with adequate training data. Another limiting factor is that all of the ultrasound recordings were acquired from the same ultrasound machine, with standardisation in the software settings. The variation in this setup in real-world scenarios could alter the final performance. However, the examination protocol for data collection and the imaging settings used were chosen to be as generic as possible. Specifically, since the transducer’s pressure on the skin during an examination is difficult to measure and standardise, the ultrasonographer avoided any pressure that could cause muscle tissue deformation by using a generous quantity of gel, thus making the examination methodology more reproducible for other operators. Regarding the imaging settings of the ultrasound device, all image optimisations were turned off to avoid emitting noise and deformation in the final representation from these settings. Again, we believe this examination setup is reproducible and can lead to some standardisation. Lastly, another limitation is the small number of sarcopenic patients participating in this study. More patients in this group could yield more reliable results, especially in the texture analysis of their muscle CSA.

Finally, in future work, we plan to investigate the automatic extraction of CSA measurement in more muscle sections and use a larger dataset of ultrasound images. Furthermore, we plan to analyse the image texture of the longitudinal ultrasound recordings to investigate whether specific patterns exist in patients suffering from sarcopenia or other neuromuscular disorders. Lastly, we plan to combine clinical data, such as gender, BMI, and the dominant side of a patient, with the features extracted from the muscle texture to fuse this information to develop a more optimised solution. This solution, which belongs to the multimodal learning field [34], will combine heterogeneous data that provide different views of the same patient to better support various clinical decisions.

## Figures and Tables

**Table 1:** Demographic characteristics of the database used in this study.

	Group 1	Group 2	Group 3	Total
Subjects	116	78	16	210
Examinations	155	78	20	253
Age (years)	25.3 ± 4.9	75.3 ± 6.6	77.3 ± 6.4	47.8 ± 15.8
Sex (M/F)	49/67	17/61	10/6	76/134
BMI (kg/m^2^)	22.9 ± 2.7	28.8 ± 4.6	25.5 ± 4.0	25.3 ± 3.6

**Table 2:** Overall segmentation results for each segmentation model. The results are reported in mean ± std.

	Precision	Recall	DSC	IoU	HD95
TRAMA	0.43 ± 0.39	0.51 ± 0.44	0.46 ± 0.40	0.39 ± 0.36	-
UNet	0.95 ± 0.05	0.95 ± 0.05	0.95 ± 0.04	0.90 ± 0.06	12.36 ± 8.85
Att-UNet	0.95 ± 0.05	0.96 ± 0.04	0.95 ± 0.03	0.91 ± 0.05	10.69 ± 7.30
UNet++	0.95 ± 0.04	0.96 ± 0.04	0.95 ± 0.03	0.91 ± 0.05	10.60 ± 7.42
UNetXt	0.95 ± 0.05	0.95 ± 0.04	0.95 ± 0.03	0.91 ± 0.05	10.96 ± 7.65
TMUNet	0.95 ± 0.04	0.96 ± 0.04	0.96 ± 0.03	0.92 ± 0.05	10.09 ± 6.86

**Table 3:** Comparison results between the manual and automatic muscle CSA measurements.

	Manual	Automatic	RMSE	HD95 (mm)	ICC	Pearson Coeff
CSA	764.50 ± 213.69 (mm^2^)	773.85 ± 210.16 (mm^2^)	38.15 ± 36.93 (mm^2^)	2.20 ± 1.44	0.97	0.97
ECHO	59.12 ± 19.14	59.39 ± 19.07	0.88 ± 0.89	-	0.99	0.99

**Table 4:** Comparison results between the manual and automatic CSA measurements per muscle.

	CSA (mm^2^)			ECHO		
	Operator	Automatic	RMSE	Operator	Automatic	RMSE
**T.A.**	728.75 ± 193.68	742.62 ± 199.64	44.05 ± 39.85	70.29 ± 18.67	70.48 ± 18.64	0.85 ± 0.86
**R.F.**	763.19 ± 231.26	776.87 ± 230.11	32.86 ± 38.14	44.46 ±15.77	44.78 ± 15.83	1.00 ± 0.99
**GCM**	752.72 ± 168.77	757.69 ± 168.02	36.00 ± 32.50	57.34 ± 13.88	57.57 ± 13.87	0.94 ± 0.98
**B.B.**	813.57 ± 244.37	818.16 ± 239.53	40.05 ± 36.66	64.44 ± 17.48	64.53 ± 17.37	0.73 ± 0.69

**Table 5:** A comparison between the manual and automatic CSA/ECHO measurements per group.

	CSA (mm^2^)			ECHO		
	Operator	Automatic	RMSE	Operator	Automatic	RMSE
**Group 1**	833.54 ± 198.68	841.06 ± 195.81	34.90 ± 33.03	52.15 ± 15.50	52.42 ± 15.50	0.70 ± 0.67
**Group 2**	660.29 ± 192.15	674.77 ± 190.93	42.91 ± 42.50	69.00 ± 19.60	69.11 ± 19.51	1.24 ± 1.18
**Group 3**	628.23 ± 174.40	632.25 ± 163.03	47.61 ± 41.14	75.32 ± 16.31	76.14 ± 16.23	1.33 ± 1.00

**Table 6:** Overall results of textural analysis of the groups for the manually and the automatically extracted CSA texture.

	Precision	Recall	F1 Score
Manual	0.84	0.86	0.84
Automatic	0.83	0.85	0.84

**Table 7:** Overall results of textural analysis of the young vs. elderly subjects for the manually and the automatically extracted CSA texture.

	Precision	Recall	F1 Score
Manual	0.94	0.94	0.94
Automatic	0.94	0.94	0.94

## Data Availability

Not applicable.

## References

[1] Hammond K., Mampilly J., Laghi F.A., Goyal A., Collins E.G., McBurney C., Jubran A., Tobin M.J. (2014). Validity and Reliability of Rectus Femoris Ultrasound Measurements: Comparison of Curved-Array and Linear-Array Transducers. J. Rehabil. Res. Dev..

[2] Seymour J.M., Ward K., Sidhu P.S., Puthucheary Z., Steier J., Jolley C.J., Rafferty G., Polkey M.I., Moxham J. (2009). Ultrasound Measurement of Rectus Femoris Cross-Sectional Area and the Relationship with Quadriceps Strength in COPD. Thorax.

[3] Sipila S., Suominen H. (1991). Ultrasound Imaging of the Quadriceps Muscle in Elderly Athletes and Untrained Men. Muscle Nerve.

[4] van Alfen N., Gijsbertse K., de Korte C.L. (2018). How Useful Is Muscle Ultrasound in the Diagnostic Workup of Neuromuscular Diseases?. Curr. Opin. Neurol..

[5] Sponbeck J.K., Frandsen C.R., Ridge S.T., Swanson D.A., Swanson D.C., Johnson A.W. (2021). Leg Muscle Cross-Sectional Area Measured by Ultrasound Is Highly Correlated with MRI. J. Foot Ankle Res..

[6] Damas F., Phillips S.M., Lixandrão M.E., Vechin F.C., Libardi C.A., Roschel H., Tricoli V., Ugrinowitsch C. (2016). Early Resistance Training-Induced Increases in Muscle Cross-Sectional Area Are Concomitant with Edema-Induced Muscle Swelling. Eur. J. Appl. Physiol..

[7] Rieder F., Kösters A., Wiesinger H.-P., Dorn U., Hofstaedter T., Fink C., Seynnes O.R., Müller E. (2015). Alpine Skiing with Total Knee ArthroPlasty (ASWAP): Muscular Adaptations. Scand. J. Med. Sci. Sports.

[8] Seymore K.D., Domire Z.J., DeVita P., Rider P.M., Kulas A.S. (2017). The Effect of Nordic Hamstring Strength Training on Muscle Architecture, Stiffness, and Strength. Eur. J. Appl. Physiol..

[9] Franchi M.V., Longo S., Mallinson J., Quinlan J.I., Taylor T., Greenhaff P.L., Narici M.V. (2018). Muscle Thickness Correlates to Muscle Cross-Sectional Area in the Assessment of Strength Training-Induced Hypertrophy. Scand. J. Med. Sci. Sports.

[10] Minetto M.A., Caresio C., Menapace T., Hajdarevic A., Marchini A., Molinari F., Maffiuletti N.A. (2016). Ultrasound-Based Detection of Low Muscle Mass for Diagnosis of Sarcopenia in Older Adults. PM&R.

[11] Salvi M., Caresio C., Meiburger K.M., De Santi B., Molinari F., Minetto M.A. (2019). Transverse Muscle Ultrasound Analysis (TRAMA): Robust and Accurate Segmentation of Muscle Cross-Sectional Area. Ultrasound Med. Biol..

[12] Caresio C., Salvi M., Molinari F., Meiburger K.M., Minetto M.A. (2017). Fully Automated Muscle Ultrasound Analysis (MUSA): Robust and Accurate Muscle Thickness Measurement. Ultrasound Med. Biol..

[13] Marzola F., van Alfen N., Doorduin J., Meiburger K.M. (2021). Deep Learning Segmentation of Transverse Musculoskeletal Ultrasound Images for Neuromuscular Disease Assessment. Comput. Biol. Med..

[14] Chen X., Xie C., Chen Z., Li Q. (2019). Automatic Tracking of Muscle Cross-Sectional Area Using Convolutional Neural Networks with Ultrasound. J. Ultrasound Med. Off. J. Am. Inst. Ultrasound Med..

[15] ACSAuto-Semi-Automatic Assessment of Human Vastus Lateralis and Rectus Femoris Cross-Sectional Area in Ultrasound Images. Scientific Reports. https://www.nature.com/articles/s41598-021-92387-6.

[16] Ritsche P., Wirth P., Cronin N.J., Sarto F., Narici M.V., Faude O., Franchi M.V. (2022). DeepACSA: Automatic Segmentation of Cross-Sectional Area in Ultrasound Images of Lower Limb Muscles Using Deep Learning. Med. Sci. Sports Exerc..

[17] Ronneberger O., Fischer P., Brox T., Navab N., Hornegger J., Wells W.M., Frangi A.F. (2015). U-Net: Convolutional Networks for Biomedical Image Segmentation. Medical Image Computing and Computer-Assisted Intervention 2015.

[18] Katakis S., Barotsis N., Kakotaritis A., Economou G., Panagiotopoulos E., Panayiotakis G. (2022). Automatic Extraction of Muscle Parameters with Attention UNet in Ultrasonography. Sensors.

[19] Dosovitskiy A., Beyer L., Kolesnikov A., Weissenborn D., Zhai X., Unterthiner T., Dehghani M., Minderer M., Heigold G., Gelly S. (2020). An Image Is Worth 16x16 Words: Transformers for Image Recognition at Scale 2021. arXiv.

[20] Shamshad F., Khan S., Zamir S.W., Khan M.H., Hayat M., Khan F.S., Fu H. (2022). Transformers in Medical Imaging: A Survey. arXiv.

[21] Azad R., Heidari M., Wu Y., Merhof D. (2022). Contextual Attention Network: Transformer Meets U-Net 2022. International Workshop on Machine Learning in Medical Imaging.

[22] Molinari F., Caresio C., Acharya U.R., Mookiah M.R.K., Minetto M.A. (2015). Advances in Quantitative Muscle Ultrasonography Using Texture Analysis of Ultrasound Images. Ultrasound Med. Biol..

[23] Katakis S., Barotsis N., Kastaniotis D., Theoharatos C., Tsiganos P., Economou G., Panagiotopoulos E., Fotopoulos S., Panayiotakis G. (2019). Muscle Type and Gender Recognition Utilising High-Level Textural Representation in Musculoskeletal Ultrasonography. Ultrasound Med. Biol..

[24] Katakis S., Barotsis N., Kastaniotis D., Theoharatos C., Tsourounis D., Fotopoulos S., Panagiotopoulos E. Muscle Type Classification on Ultrasound Imaging Using Deep Convolutional Neural Networks. Proceedings of the 2018 IEEE 13th Image, Video, and Multidimensional Signal Processing Workshop (IVMSP).

[25] Yi J., Shin Y., Hahn S., Lee Y.H. (2022). Deep Learning Based Sarcopenia Prediction from Shear-Wave Ultrasonographic Elastography and Gray Scale Ultrasonography of Rectus Femoris Muscle. Sci. Rep..

[26] Yang K.-C., Liao Y.-Y., Chang K.-V., Huang K.-C., Han D.-S. (2020). The Quantitative Skeletal Muscle Ultrasonography in Elderly with Dynapenia but Not Sarcopenia Using Texture Analysis. Diagnostics.

[27] Selvaraju R.R., Cogswell M., Das A., Vedantam R., Parikh D., Batra D. (2020). Grad-CAM: Visual Explanations from Deep Networks via Gradient-Based Localization. Int. J. Comput. Vis..

[28] Barotsis N., Galata A., Hadjiconstanti A., Panayiotakis G. (2020). The Ultrasonographic Measurement of Muscle Thickness in Sarcopenia. A Prediction Study. Eur. J. Phys. Rehabil. Med..

[29] Barotsis N., Tsiganos P., Kokkalis Z., Panayiotakis G., Panagiotopoulos E. (2020). Reliability of Muscle Thickness Measurements in Ultrasonography. Int. J. Rehabil. Res..

[30] Oktay O., Schlemper J., Le Folgoc L., Lee M., Heinrich M., Misawa K., Mori K., McDonagh S., Hammerla N.Y., Kainz B. (2018). Attention U-Net: Learning Where to Look for the Pancreas. arXiv.

[31] Zhou Z., Siddiquee M.M.R., Tajbakhsh N., Liang J. (2018). UNet++: A Nested U-Net Architecture for Medical Image Segmentation. Deep Learning in Medical Image Analysis and Multimodal Learning for Clinical Decision Support.

[32] Valanarasu J.M.J., Patel V.M. (2022). UNeXt: MLP-Based Rapid Medical Image Segmentation Network. arXiv.

[33] Bertels J., Eelbode T., Berman M., Vandermeulen D., Maes F., Bisschops R., Blaschko M. (2019). Optimizing the Dice Score and Jaccard Index for Medical Image Segmentation: Theory & Practice. International Conference on Medical Image Computing and Computer-Assisted Intervention.

[34] Cui C., Yang H., Wang Y., Zhao S., Asad Z., Coburn L.A., Wilson K.T., Landman B.A., Huo Y. (2022). Deep Multi-Modal Fusion of Image and Non-Image Data in Disease Diagnosis and Prognosis: A Review 2022. arXiv.

